# The Insoluble Protein Deposit (IPOD) in Yeast

**DOI:** 10.3389/fnmol.2018.00237

**Published:** 2018-07-12

**Authors:** Stephanie Rothe, Abaya Prakash, Jens Tyedmers

**Affiliations:** Department of Medicine I and Clinical Chemistry, Heidelberg University Hospital, Heidelberg, Germany

**Keywords:** yeast (*Saccharomyces cerevisiae*), amyloid aggregates, neurodegenerative disease, insoluble protein deposit (IPOD), phagophore assembly site (PAS), actin, vesicular transport, Atg9 vesicles

## Abstract

The appearance of protein aggregates is a hallmark of several pathologies including many neurodegenerative diseases. Mounting evidence suggests that the accumulation of misfolded proteins into inclusions is a secondary line of defense when the extent of protein misfolding exceeds the capacity of the Protein Quality Control System, which mediates refolding or degradation of misfolded species. Such exhaustion can occur during severe proteotoxic stress, the excessive occurrence of aggregation prone protein species, e.g., amyloids, or during ageing. However, the machinery that mediates recognition, recruitment and deposition of different types of misfolded proteins into specific deposition sites is only poorly understood. Since emerging principles of aggregate deposition appear evolutionarily conserved, yeast represents a powerful model to study basic mechanisms of recognition of different types of misfolded proteins, their recruitment to the respective deposition site and the molecular organization at the corresponding site. Yeast possesses at least three different aggregate deposition sites, one of which is a major deposition site for amyloid aggregates termed Insoluble PrOtein Deposit (IPOD). Due to the link between neurodegenerative disease and accumulation of amyloid aggregates, the IPOD is of particular interest when we aim to identify the molecular mechanisms that cells have evolved to counteract toxicity associated with the occurrence of amyloid aggregates. Here, we will review what is known about IPOD composition and the mechanisms of recognition and recruitment of amyloid aggregates to this site in yeast. Finally, we will briefly discuss the possible physiological role of aggregate deposition at the IPOD.

## Introduction

The Protein Quality Control System, comprising molecular chaperones and proteolytic machineries, ensures that proteins reach and maintain their native state. It recognizes misfolded species and either reverts them to the native state or eliminates them (Bukau et al., 2006; Hartl and Hayer-Hartl, 2009). However, when the generation of misfolded proteins exceeds the capacity of those systems, they accumulate and can coalesce into aggregates. Aggregates can be structurally very diverse. They range from more amorphously appearing aggregates with a low degree of structured elements to those with a high degree of structure such as amyloid aggregates (Kikis et al., 2010; Tyedmers et al., 2010a; Hipp et al., 2014). Amyloids are highly ordered, insoluble fibrous aggregates with a very high content of β-strands being oriented perpendicularly to the fibril axis. Their occurrence is a hallmark of several fatal neurodegenerative diseases (Knowles et al., 2014). It is currently still under debate why amyloid aggregates can become detrimental to the cell, but it was suggested that one determinant is the capacity of the aggregates to promote aberrant protein interactions that can capture other essential cellular proteins (Olzscha et al., 2011; Park et al., 2013; Hipp et al., 2014). Thus, mounting evidence supports the hypothesis that the sequestration of aggregates including amyloids into specialized deposition sites is a key defensive strategy for protecting the cell from harmful interactions. In case of amyloidogenic proteins, sequestration may limit templated conversion of other protein molecules into the amyloid form (Kopito, 2000; Arrasate et al., 2004; Tanaka et al., 2004; Tyedmers et al., 2010a; Olzscha et al., 2011; Holmes et al., 2014). Not surprisingly then, aggregate deposition sites have evolved very early during evolution and hence exist in simple eukaryotes such as yeast as well as in humans (Kaganovich et al., 2008; Tyedmers et al., 2010a; Sontag et al., 2014; Miller et al., 2015b).

**Figure 1:**
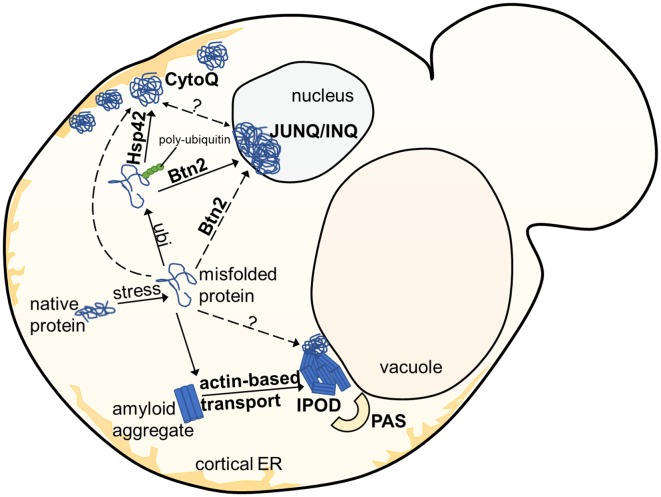
Overview of aggregate deposition sites in the yeast *S. cerevisiae*. Upon exposure to stress, misfolded or damaged proteins are targeted for either degradation or refolding, aided by molecular chaperones. Soluble protein aggregates are either targeted to the JUNQ/INQ compartment by the nuclear sorting factor Btn2, or to the peripherally localized Q-Bodies/CytoQ by the cytosolic Hsp42. Amyloidogenic aggregates accumulate predominantly at the perivacuolar insoluble protein deposit (IPOD) site adjacent to the Phagophore Assembly Site (PAS), targeted by an actin-based transport machinery, which has not yet been completely elucidated.

**Figure 2:**
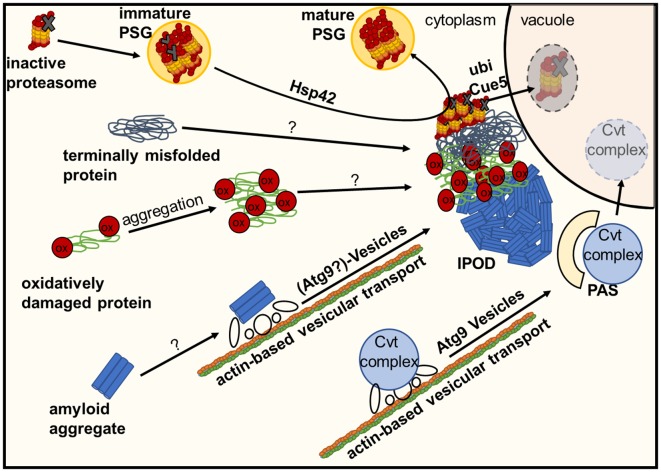
Deposition of damaged or inactive proteins, amyloids or protein complexes at the IPOD. Inactive proteasomes associated with Proteasome Storage Granules (PSGs) are known to accumulate at the IPOD in a Hsp42-dependent manner. Amyloid aggregates are targeted there by an actin-based transport machinery which overlaps with the recruitment machinery for vacuolar hydrolase precursors and their specific receptor (Cvt complex) to the pre-autophagosomal structure (PAS) via Atg9 vesicles, where these precursors are packaged into cytoplasm-to-vacuole vesicles for delivery to the lumen of the vacuole. It is hypothesized that large terminally misfolded proteins and oxidatively damaged proteins also accumulate at the IPOD in an as yet unknown manner.

In yeast, several often spatially separated deposition sites have been described (Figure 1). Those comprise: (i) the “JUxtaNuclear Quality Control Compartment (JUNQ)” (Kaganovich et al., 2008); (ii) the “IntraNuclear Quality Control Compartment (INQ)” (Gallina et al., 2015; Miller et al., 2015a); (iii) the “Insoluble PrOtein Deposit (IPOD)” (Kaganovich et al., 2008); (iv) peripheral aggregates (Specht et al., 2011; Malinovska et al., 2012; Shiber et al., 2013); (v) stress foci (Spokoini et al., 2012); and (vi) “Q-Bodies” (Escusa-Toret et al., 2013). The latter three structures were suggested to represent the same structure. It was simply discovered and named differently by different laboratories. It was therefore proposed to rename these structures as “CytoQ” (Miller et al., 2015a). The JUNQ and INQ compartments are formed under similar conditions by similar model substrates but differ in their cellular localization. While the JUNQ displays a perinuclear localization in an indentation of the nuclear envelope (Kaganovich et al., 2008), the INQ is an intranuclear site (Gallina et al., 2015; Miller et al., 2015a). It is currently under debate whether they represent identical or independent structures (Miller et al., 2015b; Hill et al., 2017; Sontag et al., 2017). Specific nuclear proteins have been identified to accumulate strictly at the INQ, which could be a future tool to test whether JUNQ and INQ are identical or different structures (Gallina et al., 2015). CytoQ, JUNQ and INQ appear predominantly during proteotoxic stress and harbor misfolded cytosolic and nuclear proteins that are more soluble and exchange rapidly with the surrounding cellular environment, whereas the IPOD seems to harbor predominantly less soluble, terminally aggregated misfolded proteins. The IPOD forms also under non-stress conditions and is described primarily as a depository for amyloid aggregates. However, it can also harbor non-amyloid substrates (Kaganovich et al., 2008; Sontag et al., 2014; Miller et al., 2015b; Hill et al., 2017). Figure 2 gives an overview of the different substrate classes deposited at the IPOD. Since especially amyloids are associated with many late-onset neurodegenerative diseases, we will focus on the IPOD, and refer to other review articles for more detailed descriptions on JUNQ, INQ and CytoQ (Sontag et al., 2014, 2017; Miller et al., 2015b; Hill et al., 2017).

## Composition of the Insoluble Protein Deposit (IPOD)

The perivacuolar IPOD (Kaganovich et al., 2008; Tyedmers et al., 2010b) is located directly adjacent to the pre-autophagosomal structure (PAS; Suzuki and Ohsumi, 2010) where the cell initiates biogenesis of autophagosomes and Cytoplasm-to-vacuole targeting (Cvt) vesicles. The IPOD was described first by Frydman and co-workers (Kaganovich et al., 2008) as a deposition site for terminally aggregated proteins. Substrate proteins accumulating here comprise amorphously aggregated, terminally misfolded proteins such as ubc9ts and VHL on the one hand and amyloidogenic proteins on the other hand. Ubc9ts is a thermosensitive variant of the SUMO-conjugating enzyme Ubc9 (Tongaonkar et al., 1999) whereas VHL is the heterologously expressed “Von Hippel-Lindau tumor supressor (VHL)” that fails to properly fold in yeast (McClellan et al., 2005). It should be noted that these model substrates partitioned not only to the IPOD, but also to the JUNQ, INQ and CytoQs (Kaganovich et al., 2008; Specht et al., 2011; Escusa-Toret et al., 2013; Miller et al., 2015a), which makes it difficult to use them as a clear-cut marker for the IPOD. This is even more relevant when one considers that CytoQs, whose formation is strictly dependent on Hsp42, present initially upon stress as multiple foci that coalesce later into very few larger ones, and under impaired proteasomal activity even into a single perivacuolar deposition that could represent the IPOD (Spokoini et al., 2012). In contrast, amyloidogenic proteins appear to be targeted exclusively to the IPOD and are often used to define a single perivacuolar inclusion as IPOD (Kaganovich et al., 2008; Tyedmers et al., 2010b; Malinovska et al., 2012). These include the yeast prions Rnq1, Ure2 and Sup35. They accumulate at the IPOD constitutively without any external stress when overproduced (Kaganovich et al., 2008; Tyedmers et al., 2010b; Saibil et al., 2012). Additional potential prion proteins (Alberti et al., 2009) that often form a single inclusion resembling the IPOD are Mot3, Lsm4 and Nrp1 (Winkler et al., 2012). Moreover, the amyloid forming “huntingtin exon1 with expanded polyglutamine and polyproline domains (Htt103Q)” is deposited at this site (Kaganovich et al., 2008; Kryndushkin et al., 2012). Additional substrates for the IPOD may include proteins that are particularly sensitive to the oxidative modification of carbonylation. Since they were found to partially co-localize with the IPOD after oxidative stress, it was suggested that they were deposited here due to oxidative damage (Tyedmers et al., 2010b). Interestingly, the major cytosolic peroxiredoxin in yeast, Tsa1, was found to be necessary to recruit Hsp70 chaperones and Hsp104 to H_2_O_2_-induced aggregates accumulating in inclusions such as JUNQ- and IPOD (Hanzen et al., 2016), further supporting a possible role of the IPOD in handling protein aggregates during oxidative stress. Furthermore, GFP fusions of proteins that are known to form inclusions in patients with amyotrophic lateral sclerosis (ALS), namely optineurin (OPTN)-GFP and an N-terminal GFP fusion of Fus1, were found to often form single inclusions co-localizing with the bonafide IPOD substrates Rnq1-GFP and Htt103Q (Kryndushkin et al., 2012). These substrates did not form amyloids in the corresponding study. In addition, it has also been reported that proteasome storage granules (PSGs) associate transiently with the IPOD upon their formation under environmental stress conditions (Peters et al., 2015, 2016). PSGs initially contain functional proteasomes as well as damaged ones. However, during transient co-localization with the IPOD, the damaged proteasomes are sorted from the PSGs to the IPOD in an Hsp42-dependent manner, while the mature PSGs, containing only functional proteasomes, dissociate from the IPOD. The proteasomes remaining at the IPOD are further heavily ubiquitinated and removed from the cell by a selective autophagic mechanism termed proteaphagy, which requires the ubiquitin receptor Cue5 (Peters et al., 2015, 2016; Marshall et al., 2016).

Thus, the fate of defective proteasomes that accumulate only transiently at the IPOD is turnover by proteaphagy. For other substrates, much less is known about their fate. For the amyloid forming fusion protein of the prion domain of Sup35 and GFP (PrD-GFP), it was shown in pulse-chase type of experiments that the protein slowly decays from the IPOD in an Hsp104 and proteasome dependent manner. Although a contribution of autophagy on turnover of PrD-GFP residing at the IPOD could not be excluded, there was no direct evidence found for this (Kumar et al., 2016). Along these lines, the yeast metacaspase Mca1 was found to localize to JUNQ and IPOD-like inclusions to counteract accumulation of terminally misfolded and aggregated proteins in an Hsp104 and proteasome-dependent manner (Hill et al., 2014).

Morphologically, IPODs consisting of the prion domain of Sup35 or GFP-fusions thereof, have been studied with different electron microscopy-based methods. This revealed that the prion amyloids in the IPODs display arrays of aligned bundles of regularly spaced fibrils without any bordering structures (Kawai-Noma et al., 2010; Tyedmers et al., 2010a; Saibil et al., 2012; O’Driscoll et al., 2015). Molecular chaperones show a non-uniform distribution within the IPOD. The chaperone Hsp104 is often found in an arrangement around the periphery of the IPOD (Kaganovich et al., 2008; O’Driscoll et al., 2015). Its presence there might reflect its roles in the disaggregation or fragmentation of prion aggregates (Paushkin et al., 1996; Kryndushkin et al., 2003; Shorter and Lindquist, 2004; Satpute-Krishnan et al., 2007; Tyedmers et al., 2010b; Winkler et al., 2012). The Hsp70 chaperones Ssa1 and Ssa2 and their co-chaperone Sis1 are very abundant within the IPOD (Bagriantsev et al., 2008; Saibil et al., 2012). Furthermore, it was found that manipulations of the Hsp70 chaperone machinery caused remodeling of the aggregates in the IPOD with a higher presence of non-fibrillar amorphous structures (O’Driscoll et al., 2015). Therefore, molecular chaperones have important roles in determining the structural organization of fibrils in the IPOD.

IPOD-like inclusions also exist in mammalian cells and have been characterized as dense, immobile, stable compartments, which are not initially ubiquitinated (Kaganovich et al., 2008; Hipp et al., 2012; Weisberg et al., 2012). In yeast, JUNQ and IPOD inclusions are tethered to organelles and dependent on a functional cytoskeleton, whereas the mammalian IPOD does not appear to be specifically associated with the cytoskeleton or the microtubule organizing center (MTOC). (Ogrodnik et al., 2014). Furthermore, additional mammalian aggregate deposition sites such as the aggresome exist where amyloid aggregates accumulate. For a more detailed view on these compartments, we refer to additional reviews (Tyedmers et al., 2010a; Amen and Kaganovich, 2015; Sontag et al., 2017).

## Substrate Targeting to the IPOD

### Targeting of Non-amyloid Substrates to the IPOD

As mentioned before, the IPOD harbors different classes of substrates, but not all of them require the same targeting signals. For the aggregation prone non-amyloid substrate OPTN-GFP, the expression levels influence its targeting efficiency to the IPOD, as higher expression levels of the construct caused appearance of additional aggregate foci next to a major IPOD deposition site (Kryndushkin et al., 2012). This predicts that the capacity for recruitment to and/or accumulation of substrates at the IPOD is limited. Furthermore, the orientation of the GFP tag influenced targeting, as despite similar expression levels, a GFP-OPTN fusion was more efficiently targeted as compared to a C-terminal OPTN-GFP fusion (Kryndushkin et al., 2012). Interestingly, a similar observation was made with Fus1-GFP: whereas Fus1-GFP formed multiple cytoplasmic foci, the corresponding N-terminal GFP-Fus1 fusion was targeted efficiently to the IPOD (Kryndushkin et al., 2011, 2012). A similar dependency on the flanking regions of an IPOD substrate was observed for different variants of Htt103Q. While the protein often aggregated in one major focus in the presence of for example a flanking poly-proline stretch, its absence caused the protein to form dispersed foci in the cytoplasm (Duennwald et al., 2006; Kaganovich et al., 2008; Wang et al., 2009; Kryndushkin et al., 2012). Although the identity of the single inclusion of Htt103Q as IPOD is still under debate, together these data suggest that there are intrinsic features in the substrates that have to be accessible to allow for efficient IPOD targeting. Whether these features may represent particular substrate conformations or binding sites for specific targeting factors remain to be elucidated.

Despite this progress in understanding determining features in the substrate, knowledge about unique targeting factors for the IPOD is very limited. In this regard, it is discussed controversially whether two confirmed targeting factors for the JUNQ/INQ and Q-Bodies/CytoQ, namely Btn2 and Hsp42, may also be involved in targeting of substrates to the IPOD. For OPTN-GFP, a co-localization with Btn2 in the major deposition site was observed. However, a direct interaction between OPTN-GFP and Btn2 was not found. Furthermore, depletion of Btn2 caused only a slight reduction in OPTN-GFP targeting to the IPOD, but did not abolish it (Kryndushkin et al., 2012). For amyloids, partial co-localization with Btn2 was also observed (Alberti, 2012; Kryndushkin et al., 2012; Malinovska et al., 2012), however Btn2 deletion showed no discernible defects in localizing amyloid aggregates to the IPOD (Alberti, 2012). Therefore, Btn2 is currently considered mostly as a targeting factor for misfolded proteins to the JUNQ/INQ (Alberti, 2012; Miller et al., 2015b).

The small heat shock protein Hsp42 has been identified as a general targeting factor for the Q-Body/CytoQ compartment (Specht et al., 2011; Escusa-Toret et al., 2013; Miller et al., 2015a). Interestingly, it was also crucial for targeting of inactive proteasomes to the IPOD (Alberti, 2012; Marshall et al., 2016; Peters et al., 2016).

### Targeting of Amyloid Substrates to the IPOD

Although Hsp42 and Btn2 have been implicated in targeting of selective substrates to the IPOD, their deletion did not affect targeting of amyloid substrates to the IPOD (Specht et al., 2011; Alberti, 2012; Escusa-Toret et al., 2013). In contrast, a possible link between a Ubiquitin-Proteasome-System component, namely the E3 ubiquitin ligase Ltn1, and deposition of amyloidogenic huntingtin aggregates into single large inclusions representing the IPOD was observed. Ltn1, also known as a key factor for targeting aborted translation products for proteasomal destruction, was found to stimulate accumulation of Htt103Q with a polyproline stretch at the IPOD. It was suggested that an intermediate range of Hsf1 activity was crucial and may regulate actin cytoskeleton dynamics and thereby affect sequestration of Htt103Q aggregates through Ltn1 (Yang et al., 2016). This possible dependency of substrate recruitment on actin cytoskeleton dynamics is in agreement with another study reporting that amyloid targeting to the IPOD was strongly reduced by depletion of proteins that function in actin cable-based transport processes, such as the motor protein Myo2 and tropomyosin (Kumar et al., 2016, 2017). An intact actin cytoskeleton is also required for aggregate targeting to the JUNQ/INQ (Specht et al., 2011) and asymmetric inheritance of protein aggregates (Ganusova et al., 2006; Liu et al., 2010, 2011; Chernova et al., 2011; Song et al., 2014). On a side note, an involvement of microtubules in the formation of JUNQ/INQ and IPOD depositions was initially suggested (Kaganovich et al., 2008), but turned out to be due to an unspecific effect of the microtubule-depolymerizing drug benomyl used in these experiments (Specht et al., 2011). Taken together, a role of the actin cytoskeleton in IPOD targeting of amyloids was consistent with previous findings. In contrast, an involvement of several proteins known to act in vesicular transport and fusion events in amyloid targeting to the IPOD (Kumar et al., 2016, 2017) was initially rather surprising. In more detail, it was found that depletion of the SNARE disassembly factor Sec18, the Sec14 and Sec21 proteins as well as deletion of the dynamin-like small GTPase Vps1 prevented accumulation of model amyloids at the IPOD but resulted reversibly in multiple smaller aggregates dispersed throughout the cytoplasm, that were interpreted as transport intermediates (Kumar et al., 2016, 2017). Strikingly, the dependency on these factors for proper amyloid targeting to the IPOD mirrored the requirements for faithful targeting of preApe1 to the perivacuolar Phagophore Assembly Site (PAS; Monastyrska et al., 2009; Lynch-Day and Klionsky, 2010; Suzuki, 2013) directly adjacent to the IPOD (Kaganovich et al., 2008; Tyedmers et al., 2010b). PreApe1 is a vacuolar precursor aminopeptidase present in large oligomeric complexes. For its recruitment to the PAS, where it is packed into autophagosome-like vesicles for delivery to the vacuolar lumen, it is attached to the outside of small transport vesicles termed Atg9 vesicles that move along actin cables (He et al., 2006). Interestingly, depleting components of this actin-cable based vesicular recruitment system did not only abolish proper accumulation of preApe1 at the PAS and amyloid aggregates at the IPOD, respectively, but both substrates co-localized with each other as multiple transport intermediates. These accumulations also harbored Myo2 and Atg9, the marker protein for Atg9 vesicles. Thus, it was hypothesized that the recruitment machinery for amyloid aggregates overlaps with that for preApe1 and involves Atg9- or related vesicles that move along actin cables with the aid of Myo2 (Kumar et al., 2016, 2017). How can a possible recruitment of amyloid aggregates on (Atg9) vesicles be reconciled with the observed effects for depletion of Sec14, 18, 21 and Vps1? Atg9 vesicles originate from the Golgi and are stored in cytoplasmic reservoirs as vesicles and tubular structures that can be activated for their rapid recruitment to the PAS if needed (Mari et al., 2010; Ohashi and Munro, 2010; Yamamoto et al., 2012). Atg9 vesicle-based transport requires several SNARE proteins (Nair and Klionsky, 2011; Nair et al., 2011). Hence, depleting Sec18, which is crucial for SNARE protein function (Mayer et al., 1996), would disturb faithful recruitment of Atg9 vesicles including their cargos to the PAS or the adjacent IPOD. Sec14 is a phosphatidylinositol/phosphatidylcholine (PI/PC) transfer protein that transfers PI lipid from the ER to the Golgi, where PI is phosphorylated to generate phosphatidylinositol-4-phosphate (PI4P; Bankaitis et al., 1990; Hama et al., 1999; Grabon et al., 2015). The pool of PI4P in turn regulates the extent to which Golgi-derived secretory vesicles can be formed (Hama et al., 1999; Grabon et al., 2015). In fact, it was demonstrated that reducing PI4P formation blocks anterograde transport of Atg9 vesicles from the Golgi to the PAS (Wang et al., 2012). Vps1 is one of three dynamin-like proteins in yeast. Dynamins are involved in membrane fusion and fission events (Williams and Kim, 2014). Strikingly, the mammalian dynamin 2 (DNM2) was recently shown to be involved in the generation of Atg9 containing vesicles (Takahashi et al., 2016). Although such a role has not been confirmed for Vps1, it seems possible that Vps1 is also involved in Atg9 vesicle biogenesis in yeast. The last protein whose depletion resulted in impaired recruitment of amyloid aggregates to the IPOD was the COPI vesicle component Sec21, which was shown recently to influence dynamics of Golgi cisternae (Ishii et al., 2016). Although this is purely speculative, interfering with Sec21 function may potentially also affect Atg9 vesicle biogenesis at the Golgi.

In summary, these data indicate that vesicle-based transport along actin cables plays an important role in recruitment of amyloids to the IPOD. This rather new concept of aggregates hitchhiking vesicular transport routes was also proposed by Nystroem and co-workers when they observed that deletion or overexpression of components of the vacuole inheritance machinery or endocytic vesicles, among them Vps1 and the myosin dependent adaptor protein Vac17, impaired or enhanced, respectively, recruitment of heat induced and Hsp104 bound aggregates to a perivacuolar inclusion site reminiscent of the IPOD (Hill et al., 2016).

## Physiological Role of Aggregate Deposition Into the IPOD

### Sequestration Function

A general function of aggregate deposition into specialized deposition sites may be sequestration, either temporal, as anticipated for the JUNQ/INQ, or prolonged as thought for the IPOD (Sontag et al., 2014; Miller et al., 2015b). As shown particularly for amyloid aggregates, their deposition may reduce harmful aberrant interactions with other cellular proteins (Olzscha et al., 2011; Park et al., 2013). This sequestration function was further substantiated by the observation that the toxicity associated with accumulating amyloidogenic proteins inversely correlated with the degree of organization: deposition into few to a single inclusion such as the IPOD was associated with less toxicity as compared to accumulation into multiple aggregate foci (Duennwald et al., 2006; Wang et al., 2009; Kryndushkin et al., 2012).

### Asymmetric Inheritance of Aggregates

Protein aggregates have been identified as aging factors that are inherited asymmetrically between mother and daughter cells. This refers to both amyloid aggregates as well as non-amyloid ones. Different principles that contribute to this asymmetry emerged, which may not be mutually exclusive (Tyedmers et al., 2010a; Nyström and Liu, 2014; Hill et al., 2017; Sontag et al., 2017). One of these mechanisms comprise the deposition of aggregates into quality control compartments such as JUNQ/INQ or IPOD, which were observed to stay in mother cells during division (Kaganovich et al., 2008; Tyedmers et al., 2010b; Spokoini et al., 2012; Hill et al., 2017). Consequently, deposition contributes to achieving damage asymmetry.

### Prion Induction

Deposition of specifically prion aggregates at the IPOD may aid another function, namely prion formation and propagation (Tyedmers et al., 2008, 2010b; Tyedmers, 2012). This hypothesis was based on the observation that a specific prion induction intermediate intersected with the IPOD (Tyedmers et al., 2010b; Saibil et al., 2012). It was discussed that misfolded species of the Sup35 prion protein ([*PSI*^+^]) in the non-prion state may be targeted to the IPOD for sequestration during proteotoxic stress. At the IPOD, Sup35 molecules get into contact with their inducer prion, [*RNQ^+^*] (Tyedmers et al., 2010b; Tyedmers, 2012) known to be present here as well (Kaganovich et al., 2008), and can adopt the prion conformation. However, other studies found that conversion to the prion state does not necessarily need to take place at the IPOD, but can also happen in the cell periphery, for example through association with actin patches (Ganusova et al., 2006; Mathur et al., 2010; Chernova et al., 2011). Most recently, it was suggested that induction of the [*PSI*^+^] prion through misfolded Sup35p molecules damaged by oxidative stress requires an active actin cytoskeleton and deposition at the IPOD, whereas prion induction by overexpression of the [*PSI*^+^] prion determinant Sup35 does not have these requirements (Speldewinde et al., 2017).

## Outlook

During the past years, it became more and more obvious that protein aggregation is not a random process but represents a second line of defense. It involves a sophisticated cellular machinery that comes into play when the cellular protein quality control systems to either refold or degrade misfolded proteins are overwhelmed. In such a situation, different types of misfolded or aberrantly folded proteins are deposited into different specialized aggregate deposition sites. This implies that the cell must be able to recognize and direct different types of misfolded proteins to the respective deposition site, and recent evidence suggests that deposition at the appropriate site can be crucial for the fidelity of the cell (Weisberg et al., 2012; Wolfe et al., 2013). Deciphering the cellular machinery and involved factors that handle amyloid aggregates will shed light into their potential role in neurodegenerative diseases. This becomes even more crucial when one considers that IPOD-like structures were described in mammalian cells as well.

Moreover, IPODs that function in sequestration of misfolded proteins and amyloids, prion induction and asymmetric distribution of aggregates during cell division, might have another potential role in the yeast cell: the IPOD may also represent a sorting center for large aggregates and high molecular weight protein complexes destined for autophagic turnover. This hypothesis originates from the observation that the substrates ranging from amyloids, terminally misfolded proteins, carbonylated, oxidatively damaged proteins (Nyström, 2005) and defective and inactive proteasomes are all large structures. Furthermore, the cell accumulates large multimeric complexes of vacuolar precursor hydrolases on transit to the vacuolar lumen via the Cvt-pathway (Lynch-Day and Klionsky, 2010; Suzuki, 2013) directly next to the IPOD. Future studies have to confirm or reject such a possible role.

## Author Contributions

JT conceptualized and wrote the manuscript. SR wrote the manuscript and designed the figures. AP wrote the manuscript.

## Conflict of Interest Statement

The authors declare that the research was conducted in the absence of any commercial or financial relationships that could be construed as a potential conflict of interest.
